# A novel, lactase-based selection and strain improvement strategy for recombinant protein expression in *Kluyveromyces lactis*

**DOI:** 10.1186/1475-2859-11-112

**Published:** 2012-08-20

**Authors:** Jorrit-Jan Krijger, Jan Baumann, Melanie Wagner, Katja Schulze, Christian Reinsch, Thomas Klose, Osita F Onuma, Claudia Simon, Sven-Erik Behrens, Karin D Breunig

**Affiliations:** 1 Institute of Biology, Martin-Luther University Halle-Wittenberg, Halle 06120, Germany; 2 Institute of Biochemistry and Biotechnology, Martin-Luther University Halle-Wittenberg, Halle, 06120, Germany

## Abstract

**Background:**

The Crabtree-negative yeast species *Kluyveromyces lactis* has been established as an attractive microbial expression system for recombinant proteins at industrial scale. Its *LAC* genes allow for utilization of the inexpensive sugar lactose as a sole source of carbon and energy. Lactose efficiently induces the *LAC4* promoter, which can be used to drive regulated expression of heterologous genes. So far, strain manipulation of *K. lactis* by homologous recombination was hampered by the high rate of non-homologous end-joining.

**Results:**

Selection for growth on lactose was applied to target the insertion of heterologous genes downstream of the *LAC4* promoter into the *K. lactis* genome and found to yield high numbers of positive transformants. Concurrent reconstitution of the β-galactosidase gene indicated the desired integration event of the expression cassette, and β-galactosidase activity measurements were used to monitor gene expression for strain improvement and fermentation optimization. The system was particularly improved by usage of a cell lysis resistant strain, VAK367-D4, which allowed for protein accumulation in long-term fermentation. Further optimization was achieved by increased gene dosage of *KlGAL4* encoding the activator of lactose and galactose metabolic genes that led to elevated transcription rates. Pilot experiments were performed with strains expressing a single-chain antibody fragment (scFv_ox_) and a viral envelope protein (BVDV-E2), respectively. scFv_ox_ was shown to be secreted into the culture medium in an active, epitope-binding form indicating correct processing and protein folding; the E2 protein could be expressed intracellularly. Further data on the influence of protein toxicity on batch fermentation and potential post-transcriptional bottlenecks in protein accumulation were obtained.

**Conclusions:**

A novel *Kluyveromyces lactis* host-vector system was developed that places heterologous genes under the control of the chromosomal *LAC4* promoter and that allows monitoring of its transcription rates by β-galactosidase measurement. The procedure is rapid and efficient, and the resulting recombinant strains contain no foreign genes other than the gene of interest. The recombinant strains can be grown non-selectively in rich medium and stably maintained even when the gene product exerts protein toxicity.

## Background

*Kluyveromyces lactis* is one of few yeast species that can utilize lactose as a sole source of carbon and energy. It can be isolated from dairy products and is used at industrial scale for preparation of the enzyme lactase (β-galactosidase). *K. lactis* received considerable interest as a eukaryotic cell factory for heterologous protein production due to physiological properties that distinguish it from *Saccharomyces cerevisiae*[1-3]. In particular, the Crabtree effect, which in *S. cerevisiae* leads to the repression of respiration under aerobic conditions, is absent or less pronounced in *K. lactis* allowing for high dilution rates and high biomass yields in fermentation processes [4-6].

With the recombinant expression of prochymosin in *K. lactis*, an industrial process was established the product of which is used in cheese manufacturing [1]. The approval of *K. lactis*-derived proteins in the food industry confirms the absence of biohazard in the host organism, which is an important aspect, for example, in the development of *K. lactis*-based oral vaccines that represents a major goal of our work [7].

A *K. lactis-*based expression system is commercially available that makes use of the strong, inducible *LAC4* promoter to drive the expression of heterologous proteins [3,8]. *LAC4* is one of the two *LAC* genes, not found in *S. cerevisiae,* that are responsible for lactose assimilation. It encodes β-galactosidase and is divergently transcribed and co-regulated with the lactose permease gene *LAC12*. The *LAC* genes are located in a subtelomeric position, and polymorphic sites have been reported [9]. The *LAC4* promoter, which has been extensively studied [10-12], is subject to transcriptional activation by KlGal4 (also termed Lac9), a homologue of the prototypic *S. cerevisiae* transactivator Gal4 [12-14]*.* KlGal4 is activated by intracellular galactose derived from cytosolic lactose hydrolysis or uptake of external galactose. Activation is controlled by a feedback regulatory network consisting of the KlGal4 inhibitor, KlGal80, and the bi-functional protein KlGal1 [15,16]. KlGal1 catalyses the conversion of galactose into galactose-1-phosphate, the first step in galactose assimilation [17]. In addition to this enzymatic activity, KlGal1 functions as an inducer of KlGal4 by binding to and inactivating KlGal80 [18].

Here we describe a novel strategy for stable, heterologous gene expression in *K. lactis* that makes use of KlGal4 to drive regulated or constitutive transcription and growth on lactose to select for integration of transgenes at the *LAC* locus. In the described host-vector system, β-galactosidase activity parallels transcription of the transgene and can be used in the establishment of fermentation protocols and selection of strains with elevated transcription. In two pilot studies we demonstrate successful secretion of a single-chain antibody fragment (scFv_ox_) and intracellular production of a viral envelope protein, the E2 protein of bovine viral diarrhea virus (BVDV). Expression of viral antigens in food-grade yeast strains is a first step towards development of yeast-based oral vaccines. As described elsewhere recombinant *K. lactis* cells could be directly used for vaccination using the strategy reported here [Arnold et al, PLoS One, in press].

## Material and methods

### *K. Lactis* strains

Strain RUL1888 MATa *ura3-59*, a *ura3* derivative of CBS2359 (NRRL Y-1140), was obtained from Dr. Yde Steensma (Leiden University, NL). RUL1888-D4 was generated by one-step gene disruption replacing *LAC4* sequences between +358 and +1181 by the *ScURA3* gene. The anti-oxazolone single-chain antibody (scFv_ox_)-producing strain RULOx was generated by replacing the *ScURA3* gene with a scFv_ox_ expression cassette by homologous recombination. Strain VAK367 *metA ura3-5* was derived from CBS2359/152 [19] via two rounds of mutagenesis selecting for 5-fluoroorotic acid resistance and screening for high stability in stationary phase and killer-negative phenotype. VAK367-D4 is a *lac4::ScURA3* derivative of VAK367, analogous to RUL1888-D4. VAK367-D4-derived recombinant strains are listed in Table 1. 

**Table 1:** *Kluyveromyces lactis* strains

**Name**	**Parent**	**Relevant genotype**	**Heterologous gene**	**Reference**
RUL1888	CBS2356	*Klura3-59*	none	H.Y. Steensma (Univ. Leiden NL)
RUL1888-D4	RUL1888	*lac4::ScURA3*	*ScURA3*	this work
RULOx	RUL1888-D4	*P*_*LAC4*_*-scFv*_*ox*_	*scFv*_*ox*_^a^	this work
RULOx/D802	RULOx	*P*_*LAC4*_*-scFv*_*ox*_*Klgal80Δ2::ScURA3*	*ScURA3*	this work
VAK367	CBS 2359/152	*metA ura3-5*	none	this work
VAK367-D4	VAK367	*lac4::ScURA3*	*ScURA3*	this work
VAK726	VAK367-D4	*P*_*LAC4*_*-E2-1*_*BVDV*_	*E2-1*_*BVDV*_^*b*^	this work
VAK746	VAK367-D4	*P*_*LAC4*_*-E2-1*_*BVDV*_*Klgal80Δ2::ScURA3*	*E2-1*_*BVDV*_^*b*^	this work
VAK834	726VAK	*P*_*LAC4*_*-E2-1*_*BVDV*_ (pLI-1)	*E2-1*_*BVDV*_^*b*^	this work
VAK836	VAK726	*P*_*LAC4*_*-E2-1*_*BVDV*_ (pLI-1)_4_	*E2-1*_*BVDV*_^*b*^	this work
VAK906	VAK726	*P*_*LAC4*_*-E2opt* (pLI-1)_n_	*E2-opt*_*BVDV*_^*c*^	this work

^a^ gene encoding oxazolon specific single-chain antibody fragment.

^b^*E2-1* segment of the bovine viral diarrhea virus (strain CP7) containing the E2 ORF and 5’ and 3’ flanking sequences.

^c^ yeast codon-optimized synthetic gene corresponding to VSV-E2(MTdel), which lacks the membrane anchor and is derived from BVDV strain NADL [22].

### Plasmids

Plasmid KIp3 is an integrative *K. lactis* vector for homologous integration at the *LAC4* or *lac4::ScURA3* locus. It consists of a ColE1 derived *E. coli* vector with the ampicillin resistance gene and an expression cassette based on the *LAC4* promoter and *TEF1* terminator that are separated by a unique *Sal*I site for insertion of genes of interest. The *TEF1* terminator is followed by the *KlGAL80* promoter and the 5'-end of the *LAC4* open reading frame. The cassette was generated by fusion-PCR in multiple steps (Additional file 1). The cassette can be separated from the vector backbone by cleavage e.g. with *Hpa*I, which creates flanking regions of homology of 1 and 1.3 kbp, respectively. KIpOx is a derivative of KIp3 containing the *S.cerevisiae* alpha-factor prepro sequence fused to the *scFv*_*ox*_ sequence with a C-terminal c-myc tag (derived from plasmid pHEN1-1 [20]). KIp3-E2-1 [7] is a derivative of KIp3 containing the coding sequence of the C-terminus of BVDV-E1 (including the signalase E1/E2cleavage site) and the full-length coding sequence of the BVDV-E2 (strain BVDV CP7, nucleotides 2273–3580; [21]; inserted as a *Sal*I-*Xho*I fragment into the unique *Sal*I site. In strain VAK906 this fragment was replaced by a yeast codon-optimized synthetic gene corresponding to VSV-E2(MTdel), which lacks the membrane anchor and is derived from BVDV strain NADL [22]. pLI-1 is an *ScURA3*-based integrative vector carrying the *KlGAL4* gene (*LAC9-1* allele) [23].

### Fermentation

Fermentations were carried out in a DasGip parallel bioreactor system (DasGip AG, Jülich, Germany) with four 2 L fully equipped fermenters. For growth studies, parallel processes were operated in a batch mode with a working volume of 0.7 L. YP medium (2% peptone, 1% yeast extract) was supplemented with mineral salts, methionine, uracil, adenine (20 mg/L each), and niacin (12 mg/L). Start lactose concentration was set to 30 g/L. The temperature of the yeast culture was maintained at 30°C and the dissolved oxygen level was controlled to 30% saturation. A pH value of 5.0 was controlled during the process via addition of 2 M NaOH and 2 M H_3_PO_4_. Samples were taken from the culture broth during the process to determine OD_600_, residual lactose and ethanol concentrations. One OD_600_ unit corresponds to 0.38 g/L dry cell weight.

### Immunofluorescence microscopy

*K. lactis* strain VAK726 was grown in YPLac medium (2% peptone, 1% yeast extract, 0.5% lactose), followed by formaldehyde fixation (5% final concentration) for 3 h at 30°C. After washing the cells with sorbitol buffer (50 mM HEPES; 1 M sorbitol, pH 7.5), cell walls were degraded with cell wall degrading enzymes (zymolyase 10 mg/ml and glusulase, 1:4) for about 30 min. Protoplasts were collected by centrifugation, and resuspended in sorbitol buffer, followed by permeabilization of the cell membrane with 0.1% Triton X-100 (in sorbitol buffer). 20 μl of the suspension was applied to poly-L-lysine-coated object slides. The dried slides were washed 3 times in PBS. After blocking (3% BSA; 0.1% cold fish gelatin, 0.1% Triton X-100; 0.05% Tween20) a 90 min incubation with a monoclonal anti-E2-antibody, [WB-214 (PA2020; c.c.pro, Germany); 1:100 in blocking solution] was performed, followed by 90 min incubation with a secondary antibody (Dylight 488, Dianova; 1:100 in blocking solution). In addition, cell nuclei and plasma membranes were stained with DAPI (1 μg/ml) and DiL C18 (Invitrogen; 1:1000 in PBS), respectively. Fluorescence signals were recorded with a confocal laser scanning microscope (TCS SP5, Leica). DAPI fluorescence (absorption 358 nm; emission 461 nm) was excited with a DPSS Laser (405 nm); DyLight488 (absorption 493 nm; emission 518 nm) with an Argon Laser (488 nm) and DilC18 (absorption 549 nm; emission 565 nm) with a HeNe Laser (543 nm).

### Preparation of BSA-oxazolone

Bovine serum albumin (BSA)-oxazolone conjugate was produced according to Mäkelä et al. [24]. Briefly, 75 mg of oxazolon (4-Ethoxymethylene-2-phenyl-2-oxazolin-5-one; Sigma E-0753) was added to 20 ml of 50 mg/ml BSA (Albumin bovine Fraction V, pH 5.2, Serva 11922) in 5% (w/v) NaHCO_3_ and shaken gently for 24 h at 4°C. After centrifugation for 30 min at 30,000 x g, the supernatant was dialysed three times against 2 L of 0.15 M NaCl at 4°C with gentle stirring, each step at least 12 h. The concentration of oxazolone was calculated from the absorption at 352 nm of the dialysate, diluted with 0.15 M NaCl to give values between 0.1 and 0.6 (ε_352_ = 32,000 M^-1^ cm^-1^). After determination of the BSA concentration, the coupling ratio was calculated and lay typically between 19 and 26 moles of oxazolone per mol of BSA with a final BSA concentration of 19–44 mg/ml. The dialysate was supplemented with an equal volume of glycerol and stored at −20°C in 100 μl aliquots.

### ELISA for scFv_ox_ detection

Microtiter plates (NUNC Maxisorp) were coated by filling the wells with 120 μl of BSA-oxazolone conjugate, diluted 1:1000 in 0.1 M NaCO_3_ pH 8.0. Filled plates were wrapped with parafilm and incubated overnight at 4°C. After incubation, the plates were emptied by vigorous shaking, filled with 120 μl of Blocking Reagent (Roche 1 112 589) diluted 1:10 in distilled water and gently shaken on a vortex for 15 sec. This procedure was repeated three times. Finally the wells were filled with 120 μl of Blocking Reagent and incubated at room temperature for 1.5 hours. Supernatants from 5 ml *K. lactis* cultures (WT and scFv_ox_-producing strains grown in YP with the indicated carbon sources at 120 rpm and 30°C for up to 110 hours) were two-fold diluted serially (undiluted to 16-fold dilution). The samples were mixed with equal volumes of Blocking Reagent and 100 μl were filled in the wells of the blocked plate, followed by 1 hour incubation at room temperature. After incubation, the plates were washed as before, filled with 100 μl of primary antibody (anti-c-myc antibody, Roche 1 667 149), diluted 1:5000 in Blocking Reagent and incubated at room temperature for 1 hour. After washing, wells were filled with 100 μl secondary antibody (anti mouse IgG-POD, Chemicon AP308P), diluted 1:2000 in Blocking Reagent and the plate was incubated at room temperature for 1 hour. After incubation, the plate was washed with substrate buffer (3.25 mM Na-perborate, 40 mM Na-citrate, 60 mM Na_2_HPO_4_, pH 4.5, stored at 4°C) and filled with 100 μl ABTS (Roche 1 112 422, 1 tablet per 50 ml of substrate buffer, stored at 4°C in darkness) per well. Reading was performed in a Tecan Sunsrise microplate reader, taking 405 nm/450 nm difference readings with 1 min intervals for 20 min with 10 sec. shaking between readings. Amounts of scFv_ox_ were determined by comparing ΔA405 for the supernatant samples to serial dilutions of a reference scFv_ox_ preparation of known concentration, purified from *Nicotiana tabacum*[25].

### Dot blots for BVDV-E2 detection

After growth for 8 h in YPLac medium, cells were harvested and protein extracts were prepared by glass bead disruption. 10 μl of protein extracts, adjusted to the same concentration, were applied to a nitrocellulose membrane (GE Healthcare). After drying and blocking (2% BSA in PBS) the membrane was incubated with an anti-E2-antibody (WB-214; 1:100) for 90 min, followed by incubation with the secondary infrared-labeled antibody (anti-ms-IRDy-800CW; LI-COR Biosciences, 1:500) for another 90 min. Fluorescence signals were detected with an Odyssey infrared imaging system (LI-COR Bioscience). Excitation and emission wavelengths were 493 nm and 518 nm, respectively.

### Northern blotting

Yeast RNA was isolated by phenol-chloroform extraction as described [26]. 5 μg total RNA was separated by agarose/formaldehyde gel electrophoresis (1% agarose; 1x MOPS buffer: 0.04 M MOPS, 10 mM sodium acetate, 1 mM EDTA, pH 7; 3.7% formaldehyde) and blotted to Hybond^TM^-N^+^ (GE Healthcare) membrane. After UV-cross-linking, the membranes were subjected to hybridization with RNA probes generated by *in vitro* transcription with T7RNA polymerase (Fermentas). Template DNAs were generated by PCR amplification of *E2* and *LAC4* sequences with one primer introducing the T7 promoter sequence. Primer sequences were T7-E2-r (TAATACG ACTCACTATAGGGAGATTAGGACTCA GCGAAGTAATC) and T-Tef REV (TGGAATTGTGAGCGGATAAC) for *E2*; LAC4-right_T7_Promoter (TAATACGACTCACTATAGGACAGCTTCATCAACCCCGTAT) and LAC4Sonde Right (CTTGAACGCTTTGAGTGCAG) for *LAC4*.

### *KlGAL4* copy number determination by qPCR

Yeast chromosomal DNA was isolated and *KlGAL4* DNA content was determined in strains VAK834 and VAK836 by qPCR using primers KlGAL4q-F (CAGCAAAG TGCAGGTGAATG) and KlGAL4q-R (AACTCCTTGCACCGTATGAC) for the *KlGAL4* gene and ACT1qPCR-FWD (ACGCTCCAAGAGCCGTCTTC) and ACT1qPCR-REV (TATCATCCCAGTTGGTAACG) for *KlACT1*, which served as the reference single copy gene. DNA amplification was recorded by SYBR-green fluorescence in an IQ5 real-time PCR system (BioRad). The copy number of *KlGAL4* in VAK906 was shown by semi-quantitative PCR to be at least two but was not quantified precisely.

## Results and discussion

### Α β-galactosidase based selection strategy for stable transformation of *K. Lactis*

Though multicopy episomal vectors are available for *K. lactis,* transformants often accumulate segregants without plasmid, even under selective conditions. This phenomenon is particularly pronounced with plasmids coding for proteins that exert a cytotoxic effect when expressed at high quantity in the yeast cell. To overcome this drawback and to generally improve *K. lactis* as a host system for heterologous protein expression, this study aimed at establishing a straight-forward procedure for the generation of stable, recombinant *K. lactis* strains. For a regulated and reproducible gene expression, these strains should carry an expression cassette integrated at a defined chromosomal position downstream of the *LAC4* promoter. Additionally, the *K. lactis* recombinants should display characteristics that enable reproducible growth in an inexpensive medium and under non-selective conditions without loss of the expression cassette.

**Figure 1:**
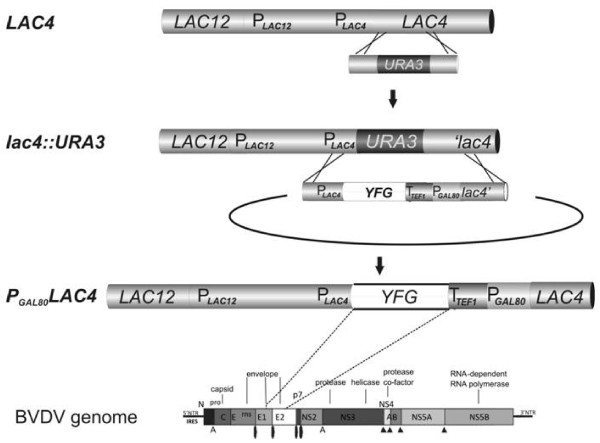
**Host-vector system for insertion of "your favorite gene (YFG)" downstream of the *LAC4 *promoter.** The *K. lactis LAC4* gene encoding β-galactosidase is divergently transcribed with the lactose permease gene (*LAC12*) and both genes are essential for growth on lactose. Disruption of *LAC4* is required to apply selection. In the *lac4::ScURA3* strain (VAK367-D4) the sequences between +358 and +1181 of the *LAC4* ORF were replaced by the *ScURA3* gene by homologous recombination. The expression cassette contains YFG fused to the *LAC4* promoter, flanked at the 3'-end by the *TEF1* terminator, the *KlGAL80* promoter and the 5'-end of *LAC4.* It can be excised from the vector backbone by cleavage with e.g. *Hpa*I. The resulting fragment can replace the *ScURA3* gene and restore the *LAC4* ORF by homologous recombination giving the *P*_*GAL80*_*-LAC4* fusion gene. Desired recombinants are selected for growth on lactose plates. Bottom: Genome structure of BVDV with E2 gene, which was inserted as an example YFG in the pilot experiment giving strain VAK726.

Along these lines, we generated a lactose*-*deficient mutant *K. lactis* strain in which the *LAC4* gene was disrupted by the *ScURA3* gene. Complementary, an integrative vector was constructed that permits the insertion of heterologous genes of choice between the *LAC4* promoter-5’UTR sequence and a transcription terminator, the latter of which is followed by the *KlGAL80* promoter and the 5’ portion of the *LAC4* ORF (Figure 1). Cleavage of this plasmid with suitable restriction enzymes generates a fragment with 1 and 1.3 kbp flanking regions that, via homologous recombination, can replace the *lac4::ScURA3* disruption cassette and restore an intact *LAC4* ORF. Regeneration of the intact *LAC4* (β-galactosidase) gene allows for growth on lactose as a sole carbon source and thus enables the selection of the desired recombinant strains. In a second screening step, the loss of the Ura^+^ phenotype serves as a marker to distinguish between a true replacement event and an unintentional insertion of the entire plasmid downstream of the *lac4::ScURA3* locus, which can also restore an intact *LAC4* gene (see below). Typically, a transformation experiment with 5 μg of cleaved plasmid DNA gave 20 to 50 Lac^+^ transformants 80% of which were Ura^-^.

In such correctly generated recombinant strains, the heterologous gene ends up at the subtelomeric *LAC* locus. It is controlled by the natural *LAC4* promoter and its long and complex control region, which co-regulates transcription of the divergent *LAC4* and *LAC12* genes [11]. Since expression of the *LAC4* ORF is now under control of the weaker *KlGAL80* promoter [27], the β-galactosidase levels produced by the recombinants are lower than that of the isogenic *LAC4* parent strain. The use of the *KlGAL80* promoter to drive *LAC4* expression accordingly serves two purposes: it avoids a tandem duplication of the *LAC4* promoters, which could lead to the loss of the insert, and it allows using β-galactosidase activity as an indicator of heterologous gene transcription (see below).

### Expression studies with a secreted model protein

The novel *K. lactis* host-vector system was next evaluated, first with a model gene encoding the single-chain antibody fragment anti-oxazolone-scFv with a C-terminal c-myc epitope (scFv_ox_; [25]). To direct the recombinant protein to the secretory pathway, the scFv_ox_ coding gene was fused to the *S. cerevisiae* prepro-alpha factor sequence. The initial host strain was derived by *LAC4* gene disruption as described [11] using strain RUL1888 (H.Y. Steensma, Univ. Leiden). After homologous recombination and selection of transformants on lactose plates, we screened for the Ura^-^ phenotype and tested for integration of the expression cassette by PCR and Southern analysis. All transformants tested had restored the *LAC4* gene, and the Ura^-^ phenotype resulted, as expected, from the desired one-step gene replacement that brought the *scFv*_*ox*_ gene under the command of the *LAC* control region. In these transformants, the *scFv*_*ox*_ gene represented YFG (“your favorite gene”; Figure 1) and was the only heterologous sequence in the *K. lactis* genome (strain RULOx). Alternatively, Lac^+^ transformants that were still Ura^+^ had restored the *LAC4* gene by integration of the entire plasmid with the expression cassette in single or multiple copies, and scFv_ox_ expression was driven by the truncated *LAC* control region present on the transforming DNA (not shown).

**Figure 2:**
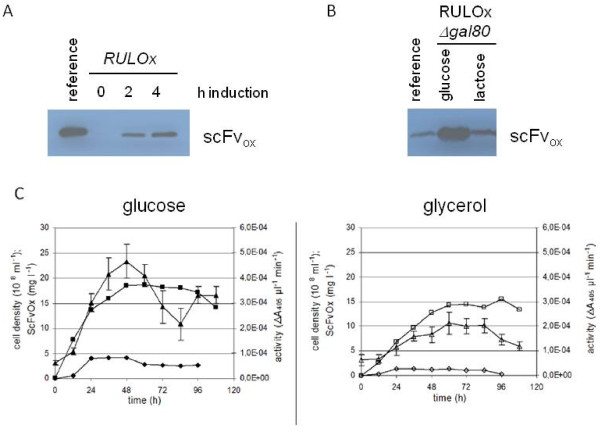
**Analysis of production of anti-oxazolone single-chain antibody fragment scFv**_**Ox**_**.A**) Inducible expression of scFv_ox_ in strain RULOx. Soluble scFv_ox_ in supernatants of YNB-cultures pregrown in glucose, 0, 2 and 4 hrs after induction by lactose was detected by Western blotting. The reference lane contains 15 ng of purified scFv_ox_. **B**) Constitutive expression of scFv_ox_ in strain RULOx/D802 (*Klgal80Δ*). Upon disruption of the *KlGAL80* gene higher levels of soluble scFv_ox_ were present in supernatants of glucose- than lactose-grown cells. The reference lane contains 5 ng of purified scFv_ox_. **C**) Long-term cultivation in glucose or glycerol medium (2% and 3% initial concentration, respectively) of the *Klgal80* mutant strain expressing scFv_ox_ (RULOx/D802 strain). Cell density (squares) is plotted with oxazolone-binding activity (triangles) and protein content (diamonds) in culture supernatant as determined by ELISA and SDS-PAGE, respectively, against the pure reference protein.

To test for expression of the antibody fragment, several recombinants were pre-grown in glucose, shifted to lactose medium (time 0), and culture supernatants were analyzed by PAGE and Western blotting. A single band at 29 kDa, corresponding approximately to the expected size for the mature scFv_ox_ (28 kDa), was detected, indicating that the prepro-signal sequence had been removed and that the protein had passed through the secretory pathway. As expected, protein expression was inducible by lactose, confirming KlGal4-regulated transcription (Figure 2A). Consistently, knock-out of the *KlGAL80* gene (strain RULOx/D802), which encodes the negative regulator of KlGal4, resulted in high expression levels of *scFv*_*ox*_ in the absence of the inducing sugars (Figure 2B).

ELISA experiments revealed that the heterologously expressed protein was delivered to the culture medium and bound the oxazolone antigen. The protein in the culture supernatant was quantified using a purified scFv_ox_myc reference protein produced in *Nicotiana tabacum* (kindly provided by Dr. U. Conrad) as a standard. The specific binding activity of the yeast derived protein was identical to the one purified from plant cells. This indicates that *K. lactis* produced correctly folded scFv_Ox_ and the affinity for the antigen was not affected by yeast specific modifications.

As described earlier, due to incomplete inactivation of the KlGal4 inhibitor under inducing conditions [27], higher *LAC4*-controlled protein levels could be obtained in the *Klgal80* mutant than in the induced wild type parent. In a first, non-optimized fermentation process of strain RULOx/D802 up to 4.4 mg/liter scFv_ox_ protein was obtained with a maximum at 48 hours of batch cultivation (Figure 2C). In glucose the level of scFv_ox_ was about 2–3 fold higher than in glycerol grown cells. In both media there was a good correlation between the accumulation of protein and binding activity. However, for unknown reasons, the binding activity dropped at late times in the batch process, while the protein appeared to be stable in Western analysis.

We had no evidence for intracellular protein accumulation or cell toxicity exerted by scFv_ox_. Compared to *Pichia pastoris*, an established yeast host system for high level single chain antibody expression [28-31], *K. lactis* did not reach the high cell densities and productivity in our non-optimized system. However, we have reason to believe that considerable improvement can be achieved by optimizing fermentation protocols and strain backgrounds. A very promising aspect of the *K. lactis* system is the low level of proteinase secretion and the solubility of the scFv_ox_.

**Figure 3:**
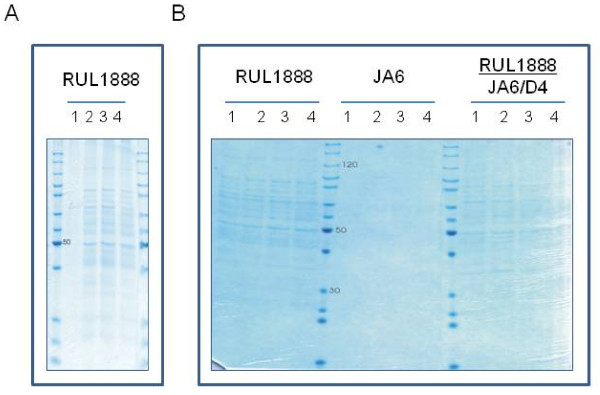
**Leakage of *K. lactis *proteins into the culture medium.A**) Strain RUL1888 was grown for 6 (lane 1), 24 (lane 2), 30 (lane 3) and 48 hrs (lane 4) in YP medium with an initial concentration of 0.04% galactose. **B**) Strain RUL1888, JA6 and a diploid strain resulting from a cross between JA6/D4 and RUL1888 were grown for 24 (lane 1), 48 (lane 2), 72 (lane 3) and 96 hrs (lane 4) in 1% galactose. Culture supernatants were applied to SDS-PAGE and gels were stained after electrophoresis by Coomassie brilliant blue.

With strain RULOx/D802 (*Klgal80*) Coomassie staining of culture supernatants revealed an unexpectedly large number of additional proteins. Moreover, β-galactosidase activity could be detected in the culture medium (not shown) at late time points of the cultivation, indicating cell lysis and release of cytoplasmic enzymes. The strain RULOx did not differ from the *Klgal80* mutant derivative in this respect (except that β-galactosidase activity was much lower). Cell lysis occurred in the stationary phase and was also detected in the non-recombinant parent strain RUL1888 (Figure 3A), ruling out that the instability was caused by expression of the heterologous protein.

In fact, among a series of *K. lactis* wild type and laboratory strains that were subsequently screened, the majority showed extracellular β-galactosidase indicating that in many *K. lactis* strains cytosolic proteins are released by cell lysis. Crossing the RUL1888 strain to JA6 [32], a stable strain with 1000- fold lower extracellular β-galactosidase activity than RUL1888, resulted in diploids that behaved like the RUL1888 parent suggesting that the cell lysis phenomenon was dominant (Figure 3B). A high correlation was observed between high β-galactosidase activity in the culture supernatant and high protein content in the growth medium. We thus used this simple enzymatic assay to generate an alternative *K. lactis* host strain for heterologous protein expression, VAK367, that did not show significant cell lysis. VAK367 originated from a Ura^-^ derivative of CBS2359/152 [19]; it had undergone two rounds of mutagenesis and surprisingly was much more stable than the grandparent CBS2359 (data not shown). Disruption of the *LAC4* gene in VAK367 using the *lac4::ScURA3* knock-out cassette (see above) did not change this property (data not shown). Thus, the resulting strain, VAK367-D4 *met ura3 lac4::ScURA3*, was subsequently used as our standard host for the integration of heterologous genes.

### Expression of the E2 envelope protein of bovine viral diarrhea virus (BVDV)

The above described selection and integration strategy was used to express several viral structural proteins. As described elsewhere, highly stable virus-like particles could be produced upon expression of murine polyoma virus VP1 in a *Klgal80* mutant strain (Simon et al., submitted). Here we report a pilot experiment on the expression of BVDV-E2 (termed E2 in this text), a highly antigenic envelope protein of the important animal pathogen bovine viral diarrhea virus (BVDV). For this purpose, the BVDV ORF encoding the C-terminus of the envelope protein E1 and the entire E2 was amplified from a cDNA of the viral isolate CP7 [21] and cloned into the KIp3 vector (E2-1 allele). An endogenous signal sequence and signal peptidase cleavage-site in the E1 portion should direct the expressed protein to the ER-membrane and enable correct processing of the E2 N-terminus [33]. After cleavage of the plasmid with *Hpa*I, which releases the expression cassette, transformation into VAK367-D4 and selection for growth on lactose were performed. The E2-1 allele was successfully integrated at the *LAC4* locus (Figure 1, bottom line) as verified by PCR and Southern blotting (data not shown). The resulting strain was named VAK726.

**Figure 4:**
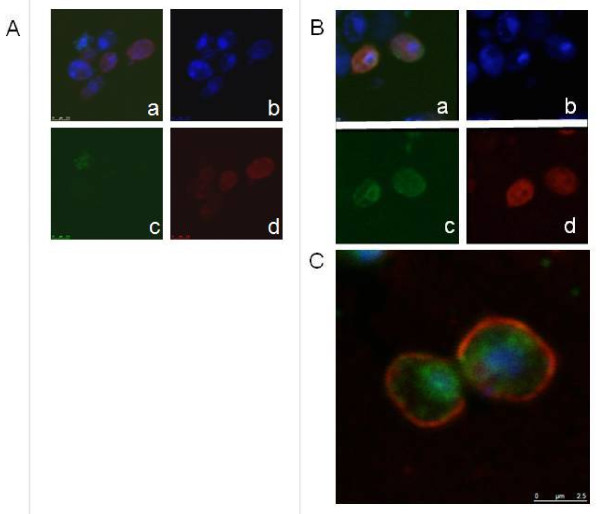
**Detection of E2 protein expression in yeast cells by immunocytology. ***K. lactis * strains VAK367 (**A**) and VAK726 (**B** and **C**), which contains the BVDV-E2 ORF in single copy under control of the *LAC4* promoter, were grown under inducing conditions (2% lactose) and analyzed by confocal microscopy after staining with DAPI (panels **b**), E2-specific antibody WB-214 (PA2020; c.c.pro, Germany) and a polyclonal secondary antibody (Dylight 488; Dianova) (panels **c**) and Dil18 (Invitrogen) for staining of the plasma membrane (panels **d**). E2 and nuclear (DAPI) staining is merged in panels **a**. **C**) Overlay of DNA, membrane-, and E2- staining reveals accumulation of E2 in perinuclear regions suggesting localization in the ER.

Expression of the E2 protein and its intracellular localization were analyzed by immunofluorescence in intact yeast cells grown on lactose medium (Figure 4). These data indicated association of the recombinant E2 with intracellular membranes, most likely of the endoplasmic reticulum (ER). Further experiments are needed to confirm E2 localisation.

### Regulated versus constitutive expression of E2

**Figure 5:**
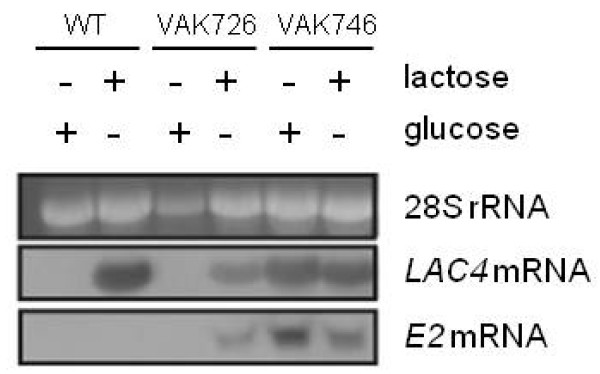
**Comparison of regulated vs. constitutive expression of *E2-1 *mRNA by Northern analysis in relation to *LAC4 *mRNA.** Total RNA was isolated from cultures grown in 0.5% glucose or lactose as indicated, separated by agarose gel electrophoresis and analyzed by hybridization with Digoxigenin (Roche)-labelled *LAC4*- or *E2*-specific RNA probes, respectively. Ethidiumbromide staining of rRNA served as a loading control (top panel).

As shown above for scFv_ox_ and elsewhere for the polyoma VP1 (Simon et al., submitted), higher protein levels were obtained in a *Klgal80* mutant conferring constitutive expression from the *LAC4* promoter. Likewise, when the *KlGAL80* gene of VAK726 was disrupted the resulting *Klgal80* mutant, strain VAK846, displayed higher *E2-1* mRNA levels than the induced parent strain with a high correlation between *E2-1* and *LAC4* mRNA levels (Figure 5). On the downside, the *Klgal80* mutation was found to reduce the fitness of the strain *K. lactis* VAK746. That is, on β-galactosidase indicator plates containing X-Gal (5-bromo-4-chloro-indolyl-galactopyranoside) white colonies were repeatedly observed with this strain. PCR analysis of these colonies revealed that they had lost the expression cassette as well as the downstream *LAC4* gene. We assumed that these variants were under positive selection due to the toxicity of the heterologous protein product when produced constitutively and at high amounts in the yeast cell. We therefore decided to stick to a regulated expression of the E2 protein.

### Increased *KlGAL4* gene dosage causes elevated expression

A major advantage of the described genomic integration strategy is that the transcription regulation of the heterologous gene and of the β-galactosidase gene follows similar rules. Considering that transcription mediated by the *LAC4* as well as the *KlGAL80* promoter is elevated at higher concentrations of KlGal4 [23,34,35], we next tested whether E2 expression could be shifted by increasing the *KlGAL4* gene dosage.

**Figure 6:**
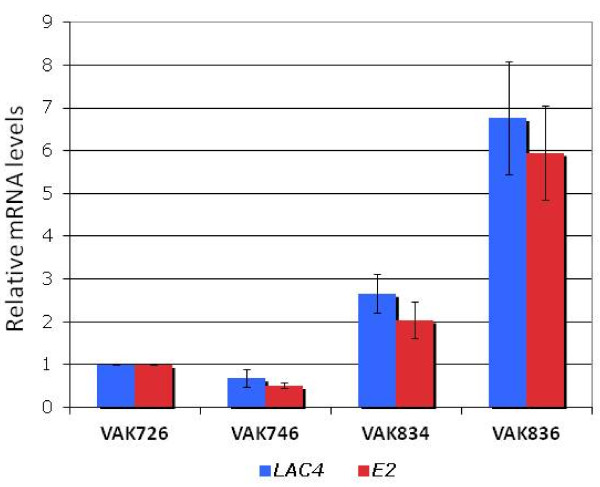
**Influence of elevated *KlGAL4 *gene dosage on *E2 *and *LAC4 *mRNA levels.** The *E2-1*-expressing strains VAK834 and VAK836 (Table 1) contain one and four tandem copies, respectively, of plasmid pLI-1 carrying *KlGAL4* in addition to the endogenous *KlGAL4* gene copy. mRNA was prepared from cells grown in 0.5% lactose and quantified by RT-qPCR using *KlACT1* as a reference.

Accordingly, a *KlGAL4* containing plasmid, pLI-1 [23], was integrated at the *KlGAL4* locus of VAK726, and Ura^+^ transformants were screened for elevated β-galactosidase activity on X-Gal-containing glucose plates. Interestingly, blue staining on glucose turned out to be a reliable marker for multiple integration of the *KlGAL4* plasmid, probably due to an imbalance between KlGal4 and KlGal80. Tandem arrangement of the *KlGAL4* gene copies was verified by PCR, and the gene copy number was determined by qPCR. Two strains containing 2 (strain VAK834) and 5 copies (strain VAK836) of the *KlGAL4* gene were further analyzed. *E2-1* mRNA levels were two and six-fold higher in strains VAK834 and VAK836, respectively, compared to VAK726 and showed a high correlation with *LAC4* mRNA levels (Figure 6). 

**Figure 7:**
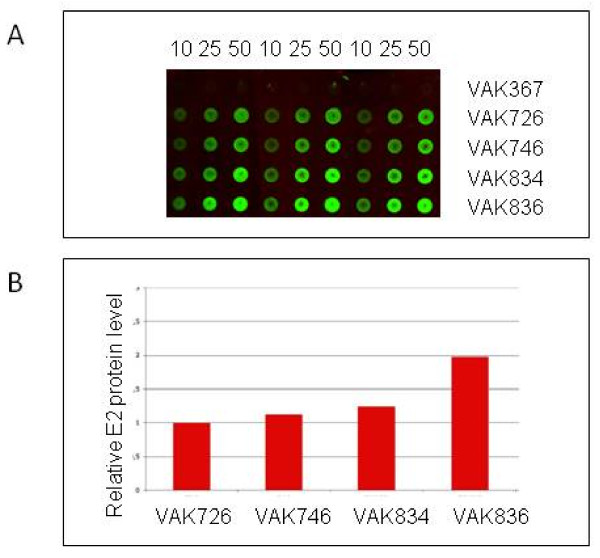
**Quantification of E2 protein levels in different congenic *K. lactis *strains.A**) Dot blots performed in triplicates with 10, 25 and 50 μg of crude cell lysates from parent strain VAK367, the E2-producing strain VAK726; the *Klgal80* mutant derivative of VAK726 (VAK746); and the two derivatives of VAK726 carrying two (VAK834) and five (VAK836) copies of the *KlGAL4* gene, respectively. Cell lysates were applied to nitrocellulose membranes and incubated with E2-specific antibody WB-214 (PA2020; c.c.pro, Germany) and polyclonal secondary antibody (Dylight 488; Dianova). **B**) Relative fluorescence signal intensities were quantified as described in Material and Methods and are given relative to strain VAK726.

To compare E2 protein levels in these yeast strains we established a dot-blot assay (Figure 7A) since denatured E2 was undetectable with the available antibodies. E2 protein present in BVDV-infected cell lysates served as a reference. Quantification of signal intensities in the dot-blot assay indicated a 20% increase of the protein level in the two-copy strain and a two-fold increase in the five-copy strain (Figure 7B). Thus, the increase in transcript levels only partially translates into higher protein levels suggesting limitations at the level of translation or protein stability. We have clear indications that codon bias is one important factor limiting translation of viral genes in *K. lactis* [Arnold et al., in revision]. The expression of yeast codon optimized synthetic genes can circumvent this problem.

### Influence of elevated *KlGAL4* gene dosage on fermentation of E2 production strains

**Figure 8:**
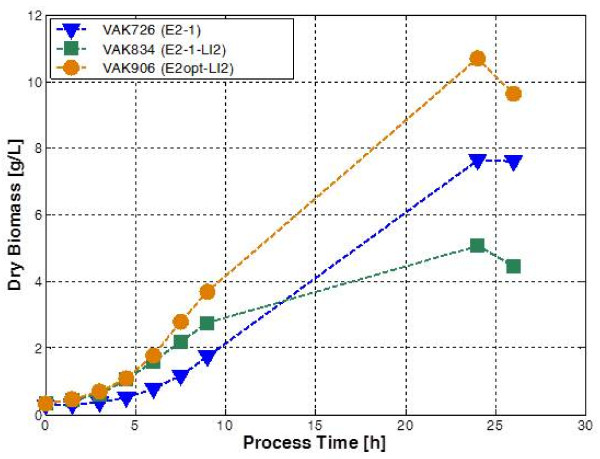
**Comparison of biomass accumulation of three E2 protein expressing strains.** Batch fermentation in supplemented YP-lactose medium (3% initial concentration) was performed in a DasGip parallel bioreactor system (DasGip AG, Jülich, Germany) under controlled conditions (see Material and Methods). Biomass accumulation is plotted against process time. VAK726 and VAK834 express the E2-1 allele of BVDV strain CP7, VAK906 contains an E2 allele from BVDV strain NADL [22]. VAK834 and VAK906 carry multiple copies of the *KlGAL4* transactivator gene.

The two E2 expressing strains VAK726 and VAK834 were compared in batch fermentation with an initial lactose concentration of 3%. The additional *KlGAL4* gene copy in VAK834 caused a significantly higher growth rate in the exponential phase (Figure 8). However, this was associated with lower yield and increased ethanol production (Table 2). At later time points strain VAK726 overtook VAK834 and after 26 hours had accumulated higher biomass levels.

**Table 2:** Comparison of the fermentation process in three different E2 expression strains

	**μ [h**^**-1**^**]**^a^	**Y**_**XS**_**[g/g]**^b^	**Y**_**EX**_**[g/g]**^c^	**X**_**f**_**[g/L]**^d^
VAK726	0.21	0.24	0.29	7.6
VAK834	0.25	0.13	1.13	5.1
VAK906	0.27	0.15	0.84	10.7

^a^μ, specific growth rate in exponential phase; ^b^Y_XS_, biomass yield from substrate; ^c^Y_EX_, ethanol per biomass; ^d^X_f_, final biomass (after 26 hrs).

Another E2 production strain, VAK906, which contains at least two *KlGAL4* gene copies, showed a different behavior. It resembles VAK834 in the initial fermentation phase but does not level off later. This indicates that the lower biomass accumulation of VAK834 is not due to the elevated KlGal4 concentration *per se.* Rather, the increased expression of the E2-1 protein appears to exert protein toxicity not found in VAK906. The *E2* gene in VAK906 was derived from another viral isolate and deviates in several aspects from the *E2-1* allele present in VAK726 and VAK834 (see Material and methods). It remains to be clarified which property favors the performance in fermentation. The results demonstrate the benefit that is eventually gained from moderate KlGal4 overexpression. However, very high levels of the transactivator exert toxicity even in a strain containing no heterologous genes [36].

## Conclusions

Here we present a new strategy to efficiently target heterologous genes to a specific location in a *K. lactis* chromosome for expression under the *K. lactis LAC4* promoter. It differs from established expression systems in the following aspects:

(i). About 80% of the transformants selected on lactose medium are Ura^-^ and have replaced the *lac4::ScURA3* disruption cassette by the transgene under the control of the complex *LAC4* promoter despite the commonly low frequency of homologous recombination in *K. lactis.*

(ii). No heterologous marker genes are used for selection of transformants; instead selection depends on reconstitution of the *K. lactis* β-galactosidase gene in a *lac4* mutant background allowing for growth on lactose. Thus, the gene of interest is the only heterologous gene in the resulting recombinant strain.

(iii). There is no need for a 5-fluoroorotic acid (FOA) selection step, which may result in cryptic mutations, to select for replacement of the *lac4::ScURA3* disruption. Since *lac4* disruptions can be easily generated using dominant markers and X-Gal indicator plates for mutant selection, any prototrophic host strain, including polyploid industrial production strains, can be engineered to meet the requirements of this host-vector system.

(iv). Expression of β-galactosidase is driven by the *KlGAL80* promoter, the activity of which parallels *LAC4* promoter activity. This allows optimizing fermentation protocols and strain improvement by simple established enzymatic assays.

(v). The recombinant strains are stable even when the gene product exerts cytotoxicity and can be grown under non-selective conditions in complex media if desired.

With the regulated production of an anti-oxazolone single-chain antibody and the bovine viral diarrhea virus envelope protein E2 we further confirmed the suitability of *K. lactis* as a eukaryotic microbial host for secretion and intracellular production of heterologous proteins that are otherwise difficult to express recombinantly. The inducible *LAC4* promoter can easily be converted into a strong constitutive promoter by gene disruption of the transcription inhibitor KlGal80 but, depending on the introduced heterologous gene, protein toxicity may become an issue. We demonstrated that inducible *LAC4* promoter activity and expression of the transgene could be further improved by integration of additional copies of the *KlGAL4* transcription activator gene. However, in the case of the BVDV-E2-1 allele cell viability dropped from >95% to <65% with elevated *KlGAL4* gene dosage (data not shown). Moreover, elevated transcription rates did not directly correlate with protein accumulation and post-transcriptional steps were uncovered as potential bottlenecks. The regulated expression under control of the *LAC4* promoter nevertheless allowed obtaining high recombinant protein expression levels.

## Competing interests

The authors declare that they have no competing interests.

## Authors contributions

JJK constructed the expression cassette and performed the scFv_ox_ experiments, JB and MW performed the E2 pilot experiment, CR analysed the cell lysis phenomenon, OFO and TK generated E2 expressing strains, KS and CS contributed to yeast fermentation, SEB designed E2 expression and co-supervised the E2 pilot study, KDB conceived the strategy, supervised the work and drafted the manuscript. All authors read and approved the final manuscript.

## Supplementary Material

Additional file 1 Construction of the scFv._**ox**_ expression cassette. Click here for file

## Author details

^1^Institute of Biology, Martin-Luther University Halle-Wittenberg, Halle 06120, Germany. ^2^Institute of Biochemistry and Biotechnology, Martin-Luther University Halle-Wittenberg, Halle 06120, Germany.
