# Supplementary material for: A novel, lactase-based selection and strain improvement strategy for recombinant protein expression in Kluyveromyces lactis
Source: Microb Cell Fact. 2012 Aug 20;11:112. doi: 10.1186/1475-2859-11-112 (PMC3520740; doi:10.1186/1475-2859-11-112)
Supplement: Additional file 1 — Construction of the scFv.ox expression cassette. [file 1475-2859-11-112-S1.docx]

**Supplementary Material**

**Construction of the scFv_ox_ expression cassette**

All PCR reactions described below were performed using *Pfu* polymerase. Where appropriate for T-A cloning, A's were added to blunt PCR fragments using *Taq* polymerase.

To place *LAC4* under control of the *KlGAL80* promoter, a *KlGAL80* promoter fragment spanning positions -643 to +4 was amplified by PCR from plasmid pKlGal80 {Zenke, 1993 951 /id} introducing an *Nco*I restriction site at the start codon using primers KG80 643_For SalI (5'-GTCGACTAGGTGATTAGCGGGGG-3'; *Sal*I site underlined) and KG80-L4 Fus_RE_NcoI (5'-GTCAGGAATAAGGCAAGCCATGGTGCCGTCC-3'; *Nco*I site underlined). This 670 bp fragment has a *Sal*I restriction site and 17 bp corresponding to *LAC4* positions +5 to +21 introduced by the primers. A 369 bp fragment of *LAC4* spanning positions +5 to +373 was amplified from plasmid pKlac2 Das {Das, 1985 66 /id} using primers L4-KG80_Fus_FOR_NcoI (5'-GGACGGCACCATGGCTTGCCTTATTCCTGAC-3'; *Nco*I site underlined) and LAC4_+373_REV (5'-GCTCGAACGACTCAATCGATTTCG-3'; *Cla*I site underlined). This 383 bp fragment has 14 bp corresponding to *KlGAL80* promoter positions -10 to +4 including the *Nco*I restriction site around the start codon and includes a *Cla*I restriction site in *LAC4* at position +354.The two fragments were fused by PCR by virtue of their 31 bp homologous ends, fusion fragments were cloned in pDrive (Qiagen, Hilden, Germany), yielding pDrive-P80LAC4-1, which was verified by sequencing. The T to G change at position +4, resulting from the introduction of the *Nco*I restriction site, led to a Ser-to-Ala exchange at position 2 of Lac4p. From P80LAC4-1, the 1003 bp P_KLGAL80_-LAC4-5' fragment was excised using *Sal*I and *Cla*I restriction sites and cloned into the 8.8 kb *Sal*I-*Cla*I vector fragment of pKlac2. The resulting ARS plasmid pKP80L4 had replaced the *LAC4* promoter by the *KlGAL80* promoter controlling expression of the *LAC4* ORF.

The unique *Sal*I restriction site preceding the *KlGAL80* promoter in plasmid pKP80L4 was used to insert the *TEF1* terminator of *Ashbya gossypii.* The terminator fragment was amplified from plasmid pUG6 {Guldener, 1996 3563 /id} using primers T-TEF_SalI_FOR (5'- AATGTCGACTCAGTACTGACAATAAAAAG-3'; *Sal*I site underlined) and T-TEF_REV (5'- ATTAAGGGTTCTCGAGAGCTCG-3'; *Xho*I site underlined). The resulting 275 bp fragment was digested with *Sal*I and *Xho*I and cloned into SalI cleaved pKP80L4 to give plasmid pKTP80L4, again with a unique *Sal*I site.

A 1.1 kb *Xho*I -*Sal*I *LAC4* promoter fragment (position -1 to -1090), amplified from plasmid pL4 {Gödecke, 1991 871 /id} using primers PL4_XhoI_FOR (5'- ATCCTCGAGTGCGGAAGAGGTAACG-3'; *Xho*I site underlined) and PL4_SalI_REV (5'- GCGGTCGACATCTTTCAGTTCTCGATG-3'; *SalI* site underlined), was cloned into the unique *Sal*I restriction site of pKTP80L4 to give episomal plasmid pKP4TP80L4. To obtain the integrative vector Kip3 the ARS1 and KARS12 sequences and the *ScTRP1* marker were removed from pKP4TP80L4 by digestion of the plasmid with *Dra*III and *Pml*I followed by isolation, blunting and recircularization. The unique *Sal*I site between *LAC4* promoter and *AgTEF1* terminator of KIp3 allows insertion of genes of interest. By cleavage with *Hpa*I bacterial vector sequences are separated from yeast sequences to be used for one-step gene replacement of the *lac4::ScURA3* disruption of appropriate *Kluyveromyces lactis* strains by homologous recombination.

To create a translational fusion of the scFv_ox_-c-myc coding sequence to the *S. cerevisiae* mating factor α1 prepro-sequence mediating classical secretion the scFv_ox_-c-myc fragment was amplified from plasmid pHEN1-1 {Hoogenboom, 1991 1487 /id} using primers Ox-PPmutfus FOR (5'-GCTTAAGCGCATGCATGACGTCAAGCTGCAGGAG-3'; *Afl*II, *Sph*I and *Aat*II sites underlined) and Ox-myc AflII REV (5'-CTTAAGCTATGCGGCCCCATTC-3'; *Afl*II site underlined). The resulting 778 bp oxazolon-c-myc fragment (Ox) was flanked by *Afl*II, *Sph*I and *Aat*II restriction sites introduced by the forward primer and an *Afl*II restriction site after the c-myc tag introduced by the reverse primer. The MFα1-prepro-sequence was amplified from plasmid pJDY15 {Abdel-Salam, 2001 3565 /id} using primers PP SalIAatII_FOR (5'-TAGTC**GACGTC**ATGAGATTTCCTTCAATTTTTAC-3'; *Sal*I site underlined, *Aat*II site bold) and PP-Ox_mutfus_REV (5'-CGTCATGCATGCGCTTAAGCAAAGATACCCCTTC-3'; *Sph*I and *Afl*II sites underlined). The resulting 267 bp fragment contains 20 bp homologous to the Ox-fragment. The two fragments were fused by PCR to give PPOx. This fragment was cloned into pCR2.1-TOPO, yielding pCR-PPOxmyc mut-1 and verified by sequencing. The PPOx fragment was excised as a *Sal*I-*Xho*I fragment and inserted into the unique *Sal*I restriction site of KIp3 to give KIpOx.
